# Supplementary material for: Detection of a Reassortant H9N2 Avian Influenza Virus with Intercontinental Gene Segments in a Resident Australian Chestnut Teal
Source: Viruses. 2020 Jan 13;12(1):88. doi: 10.3390/v12010088 (PMC7019556; doi:10.3390/v12010088)
Supplement: Supplementary file 1 [file viruses-12-00088-s001.pdf]

Detection of a reassortant H9N2 avian influenza virus with intercontinental gene segments in a resident Australian Chestnut teal

Tarka Raj Bhatta[1,2], Anthony Chamings [1,2], Jessy Vibin[1,2], Marcel Klaassen[1,3], and Soren Alexandersen[1,2,4*]

[1]Geelong Centre for Emerging Infectious Diseases, Geelong, Victoria 3220, Australia

[2]Deakin University, School of Medicine, Geelong, Victoria 3220, Australia

[3]Deakin University, Centre for Integrative Ecology, Victoria 3220, Australia

[4]Barwon Health, University Hospital Geelong, Geelong, Victoria 3220 Australia

*Corresponding Author: soren.alexandersen@deakin.edu.au; Tel.: +61 (0) 342159635 (S.A)

Received: date; Accepted: date; Published: date

Table S1. Partial sequences of the eight gene segments of AIV obtained from A/Chestnut teal/Australia/CT08.18/2018(H9N2).

Gene Segments	References used for comparisons and region calculations	Accession number	Sequence length (bp)	Region	No. of reads
PB2	A/duck/Bangladesh/26992/2015(H7N9)	KY635525	133	92-224	1
			128	340-467	1
			219	691-909	4
			421	1018-1438	10
			244	1458-1701	4
			173	1810-1982	1
PB1	A/duck/Mongolia/154/2015(H1N2)	LC121274	115	70-184	1
			164	256-419	7
			406	541-946	12
			236	981-1216	3
			238	1277-1514	2
			172	1517-1688	7
			99	1771-1869	1
			165	1872-2036	5
PA	A/Grey Teal/Victoria/GT001/2017(H9N1)	MK213327	139	71-209	1
			196	410-605	1
			140	616-755	1
			277	792-1068	3
			149	1346-1494	1
			229	1904-2132	2
HA	A/Grey Teal/Victoria/GT001/2017(H9N1)	MK213322	326	315-640	10
			396	989-1384	30
			129	1417-1545	3
			124	1585-1708	3
NP	A/RuddyTurnstone/MW02/Tas/2014(H10N8)	MH453824	278	7-284	4
			116	610-725	3
			349	856-1204	9
			105	1437-1541	2
NA	A/duck/Mongolia/154/2015(H1N2)	LC121278	261	209-469	4
			258	648-905	5
			202	914-1115	5
			210	1130-1339	5
M1-M2	A/Grey Teal/Victoria/GT001/2017(H9N1)	MK213323	264	1-264	1
			484	493-976	9
NEP-NS1	A/RuddyTurnstone/MW02/Tas/2014(H10N8)	MH453825	140	39-178	1
			123	256-378	3
			434	431-864	4

Figure S1

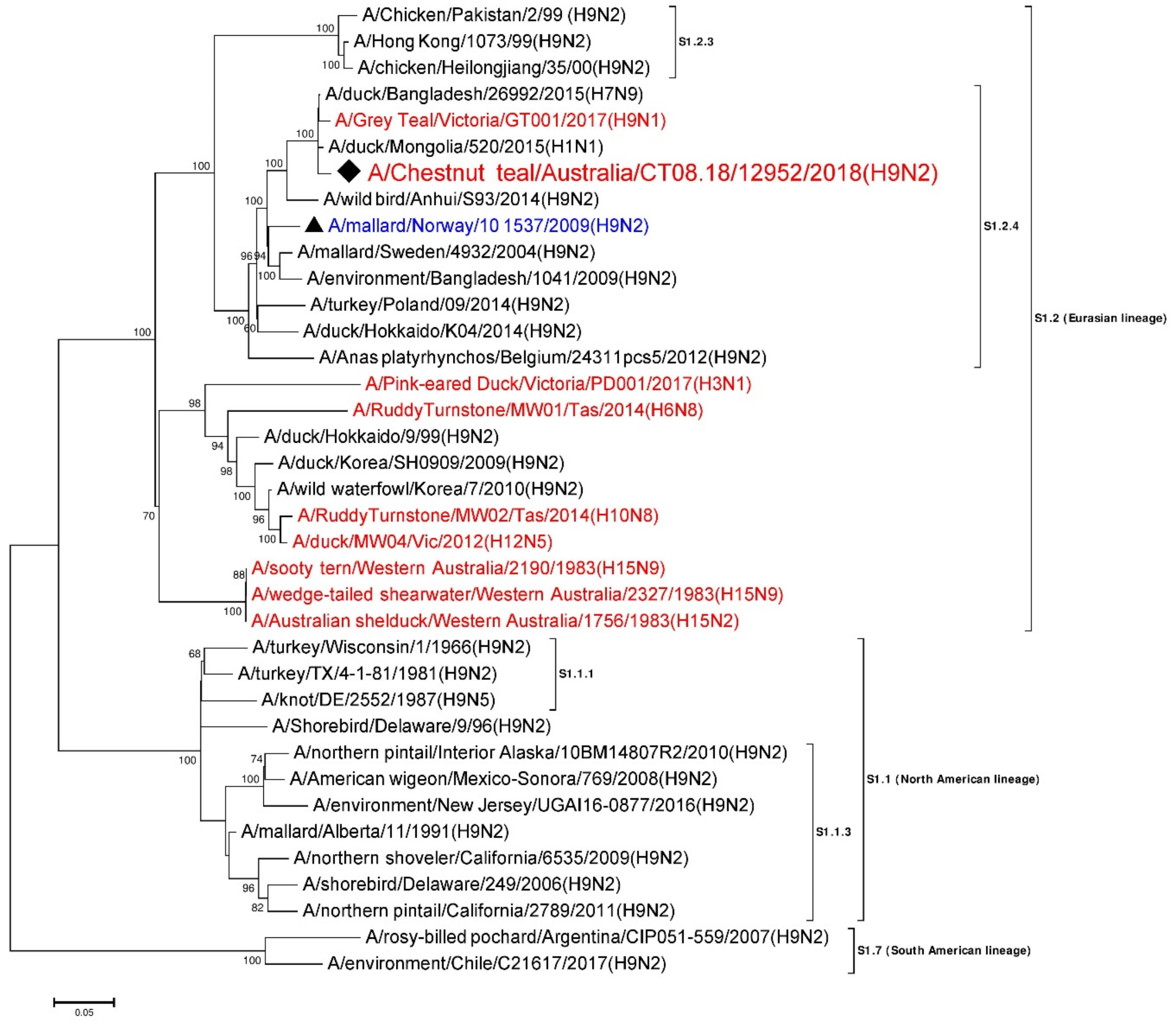
A/Chicken/Pakistan/2/99 (H9N2)
A/Hong Kong/1073/99(H9N2)
A/chicken/Heilongjiang/35/00(H9N2)
S1.2.3
A/duck/Bangladesh/26992/2015(H7N9)
A/Grey Teal/Victoria/GT001/2017(H9N1)
A/duck/Mongolia/520/2015(H1N1)
A/Chestnut teal/Australia/CT08.18/12952/2018(H9N2)
A/wild bird/Anhui/S93/2014(H9N2)
A/mallard/Norway/10 1537/2009(H9N2)
A/mallard/Sweden/4932/2004(H9N2)
A/environment/Bangladesh/1041/2009(H9N2)
A/turkey/Poland/09/2014(H9N2)
A/duck/Hokkaido/K04/2014(H9N2)
A/Anas platyrhynchos/Belgium/24311pcs5/2012(H9N2)
S1.2.4
S1.2 (Eurasian lineage)
A/Pink-eared Duck/Victoria/PD001/2017(H3N1)
A/RuddyTurnstone/MW01/Tas/2014(H6N8)
A/duck/Hokkaido/9/99(H9N2)
A/duck/Korea/SH0909/2009(H9N2)
A/wild waterfowl/Korea/7/2010(H9N2)
A/RuddyTurnstone/MW02/Tas/2014(H10N8)
A/duck/MW04/Vic/2012(H12N5)
A/sooty tern/Western Australia/2190/1983(H15N9)
A/wedge-tailed shearwater/Western Australia/2327/1983(H15N9)
A/Australian shelduck/Western Australia/1756/1983(H15N2)
A/turkey/Wisconsin/1/1966(H9N2)
A/turkey/TX/4-1-81/1981(H9N2)
A/knot/DE/2552/1987(H9N5)
S1.1.1
A/Shorebird/Delaware/9/96(H9N2)
A/northern pintail/Interior Alaska/10BM14807R2/2010(H9N2)
A/American wigeon/Mexico-Sonora/769/2008(H9N2)
A/environment/New Jersey/UGAI16-0877/2016(H9N2)
A/mallard/Alberta/11/1991(H9N2)
A/northern shoveler/California/6535/2009(H9N2)
A/shorebird/Delaware/249/2006(H9N2)
A/northern pintail/California/2789/2011(H9N2)
S1.1.3
S1.1 (North American lineage)
A/rosy-billed pochard/Argentina/CIP051-559/2007(H9N2)
A/environment/Chile/C21617/2017(H9N2)
S1.7 (South American lineage)
0.05

Figure S2

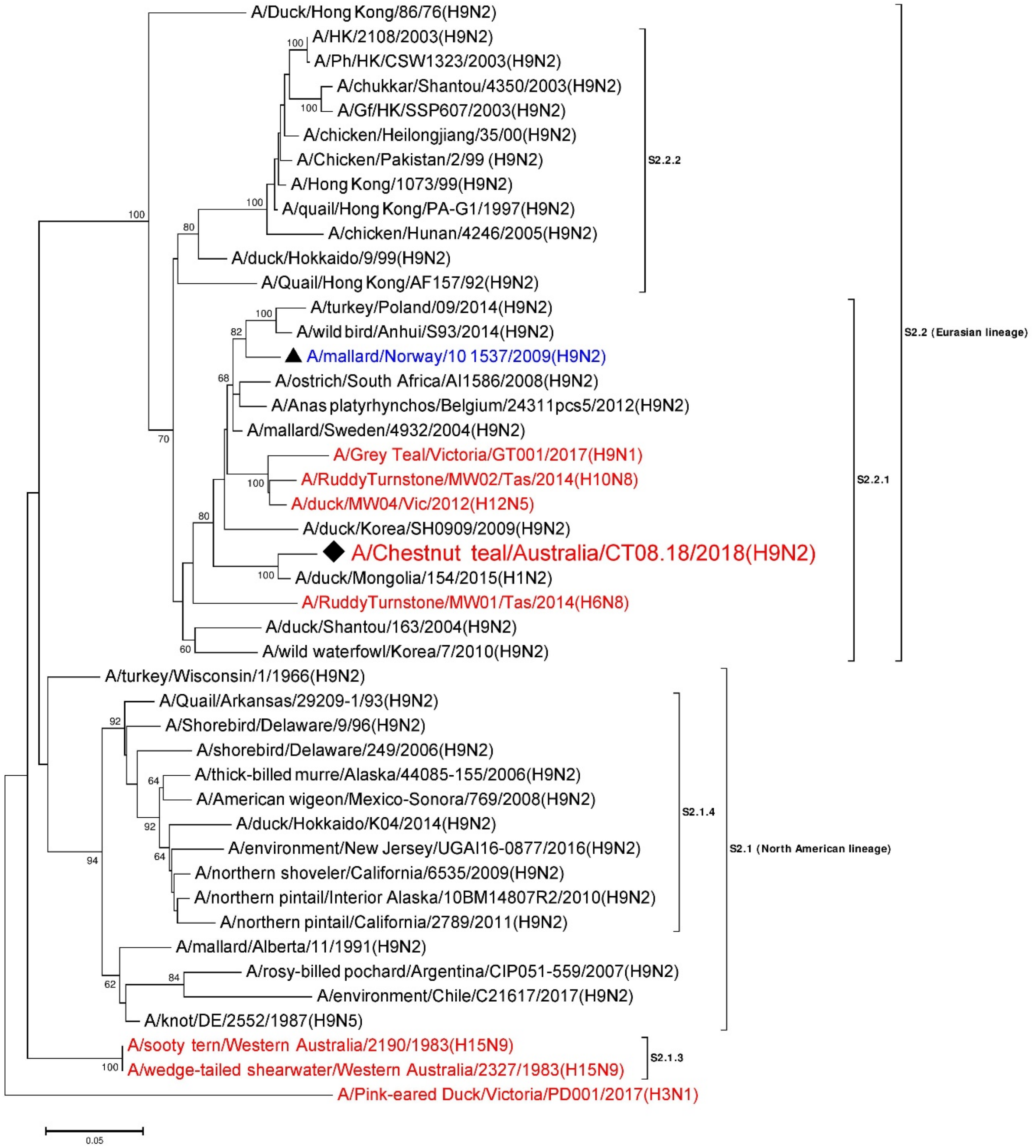
A/Duck/Hong Kong/86/76(H9N2)
A/HK/2108/2003(H9N2)
A/Ph/HK/CSW1323/2003(H9N2)
A/chukkar/Shantou/4350/2003(H9N2)
A/Gf/HK/SSP607/2003(H9N2)
A/chicken/Heilongjiang/35/00(H9N2)
A/Chicken/Pakistan/2/99 (H9N2)
A/Hong Kong/1073/99(H9N2)
A/quail/Hong Kong/PA-G1/1997(H9N2)
A/chicken/Hunan/4246/2005(H9N2)
A/duck/Hokkaido/9/99(H9N2)
A/Quail/Hong Kong/AF157/92(H9N2)
A/turkey/Poland/09/2014(H9N2)
A/wild bird/Anhui/S93/2014(H9N2)
A/mallard/Norway/10 1537/2009(H9N2)
A/ostrich/South Africa/AI1586/2008(H9N2)
A/Anas platyrhynchos/Belgium/24311pcs5/2012(H9N2)
A/mallard/Sweden/4932/2004(H9N2)
A/Grey Teal/Victoria/GT001/2017(H9N1)
A/RuddyTurnstone/MW02/Tas/2014(H10N8)
A/duck/MW04/Vic/2012(H12N5)
A/duck/Korea/SH0909/2009(H9N2)
A/Chestnut teal/Australia/CT08.18/2018(H9N2)
A/duck/Mongolia/154/2015(H1N2)
A/RuddyTurnstone/MW01/Tas/2014(H6N8)
A/duck/Shantou/163/2004(H9N2)
A/wild waterfowl/Korea/7/2010(H9N2)
A/turkey/Wisconsin/1/1966(H9N2)
A/Quail/Arkansas/29209-1/93(H9N2)
A/Shorebird/Delaware/9/96(H9N2)
A/shorebird/Delaware/249/2006(H9N2)
A/thick-billed murre/Alaska/44085-155/2006(H9N2)
A/American wigeon/Mexico-Sonora/769/2008(H9N2)
A/duck/Hokkaido/K04/2014(H9N2)
A/environment/New Jersey/UGAI16-0877/2016(H9N2)
A/northern shoveler/California/6535/2009(H9N2)
A/northern pintail/Interior Alaska/10BM14807R2/2010(H9N2)
A/northern pintail/California/2789/2011(H9N2)
A/mallard/Alberta/11/1991(H9N2)
A/rosy-billed pochard/Argentina/CIP051-559/2007(H9N2)
A/environment/Chile/C21617/2017(H9N2)
A/knot/DE/2552/1987(H9N5)
A/sooty tern/Western Australia/2190/1983(H15N9)
A/wedge-tailed shearwater/Western Australia/2327/1983(H15N9)
A/Pink-eared Duck/Victoria/PD001/2017(H3N1)
S2.2.2
S2.2.1
S2.2 (Eurasian lineage)
S2.1.4
S2.1 (North American lineage)
S2.1.3
0.05

Figure S3

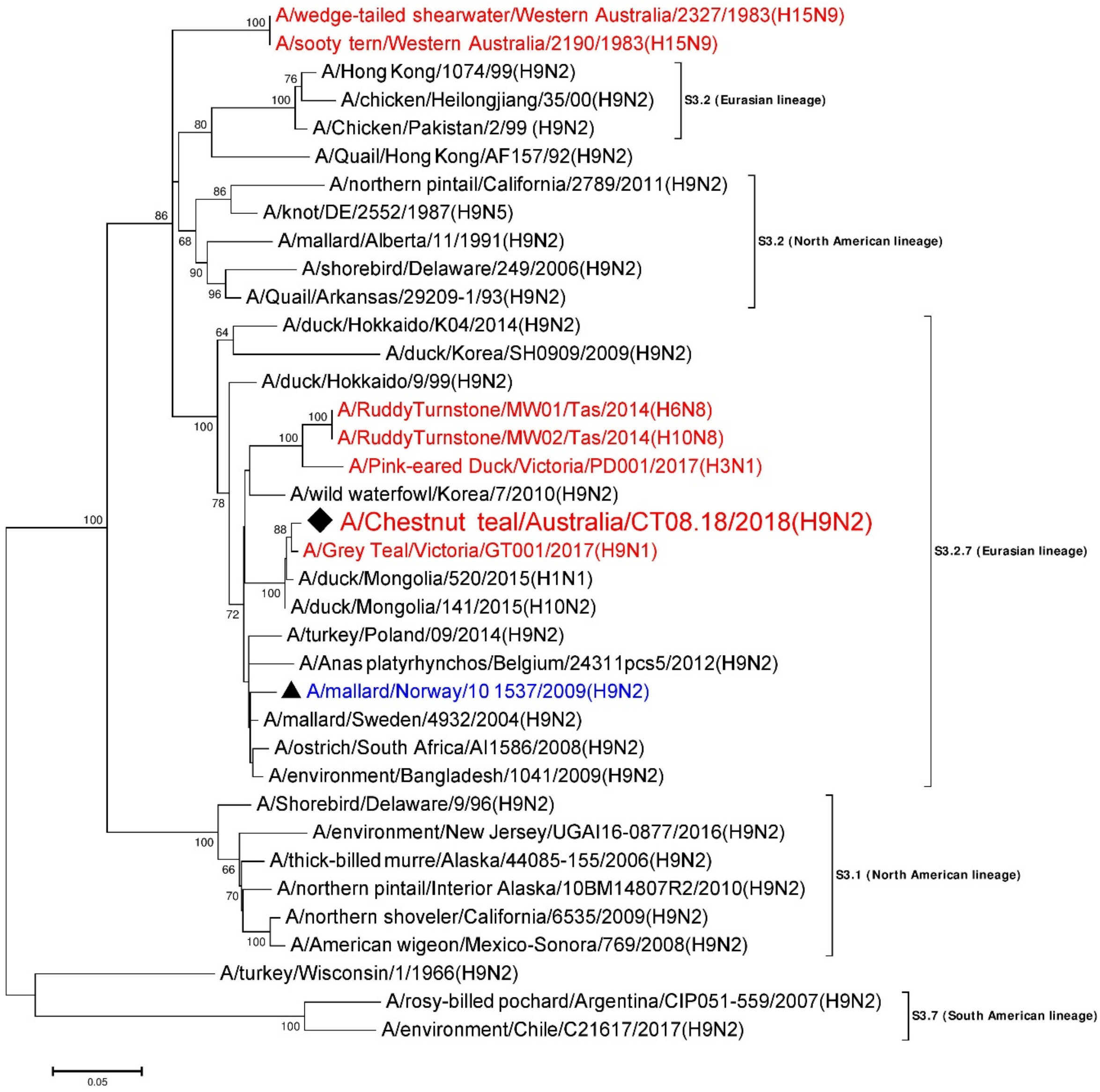

A/wedge-tailed shearwater/Western Australia/2327/1983(H15N9)
A/sooty tern/Western Australia/2190/1983(H15N9)
A/Hong Kong/1074/99(H9N2)
A/chicken/Heilongjiang/35/00(H9N2)
A/Chicken/Pakistan/2/99 (H9N2)
A/Quail/Hong Kong/AF157/92(H9N2)
S3.2 (Eurasian lineage)
A/northern pintail/California/2789/2011(H9N2)
A/knot/DE/2552/1987(H9N5)
A/mallard/Alberta/11/1991(H9N2)
A/shorebird/Delaware/249/2006(H9N2)
A/Quail/Arkansas/29209-1/93(H9N2)
S3.2 (North American lineage)
A/duck/Hokkaido/K04/2014(H9N2)
A/duck/Korea/SH0909/2009(H9N2)
A/duck/Hokkaido/9/99(H9N2)
A/RuddyTurnstone/MW01/Tas/2014(H6N8)
A/RuddyTurnstone/MW02/Tas/2014(H10N8)
A/Pink-eared Duck/Victoria/PD001/2017(H3N1)
A/wild waterfowl/Korea/7/2010(H9N2)
A/Chestnut teal/Australia/CT08.18/2018(H9N2)
A/Grey Teal/Victoria/GT001/2017(H9N1)
A/duck/Mongolia/520/2015(H1N1)
A/duck/Mongolia/141/2015(H10N2)
A/turkey/Poland/09/2014(H9N2)
A/Anas platyrhynchos/Belgium/24311pcs5/2012(H9N2)
A/mallard/Norway/10 1537/2009(H9N2)
A/mallard/Sweden/4932/2004(H9N2)
A/ostrich/South Africa/AI1586/2008(H9N2)
A/environment/Bangladesh/1041/2009(H9N2)
S3.2.7 (Eurasian lineage)
A/Shorebird/Delaware/9/96(H9N2)
A/environment/New Jersey/UGAI16-0877/2016(H9N2)
A/thick-billed murre/Alaska/44085-155/2006(H9N2)
A/northern pintail/Interior Alaska/10BM14807R2/2010(H9N2)
A/northern shoveler/California/6535/2009(H9N2)
A/American wigeon/Mexico-Sonora/769/2008(H9N2)
S3.1 (North American lineage)
A/turkey/Wisconsin/1/1966(H9N2)
A/rosy-billed pochard/Argentina/CIP051-559/2007(H9N2)
A/environment/Chile/C21617/2017(H9N2)
S3.7 (South American lineage)
0.05

Figure S4

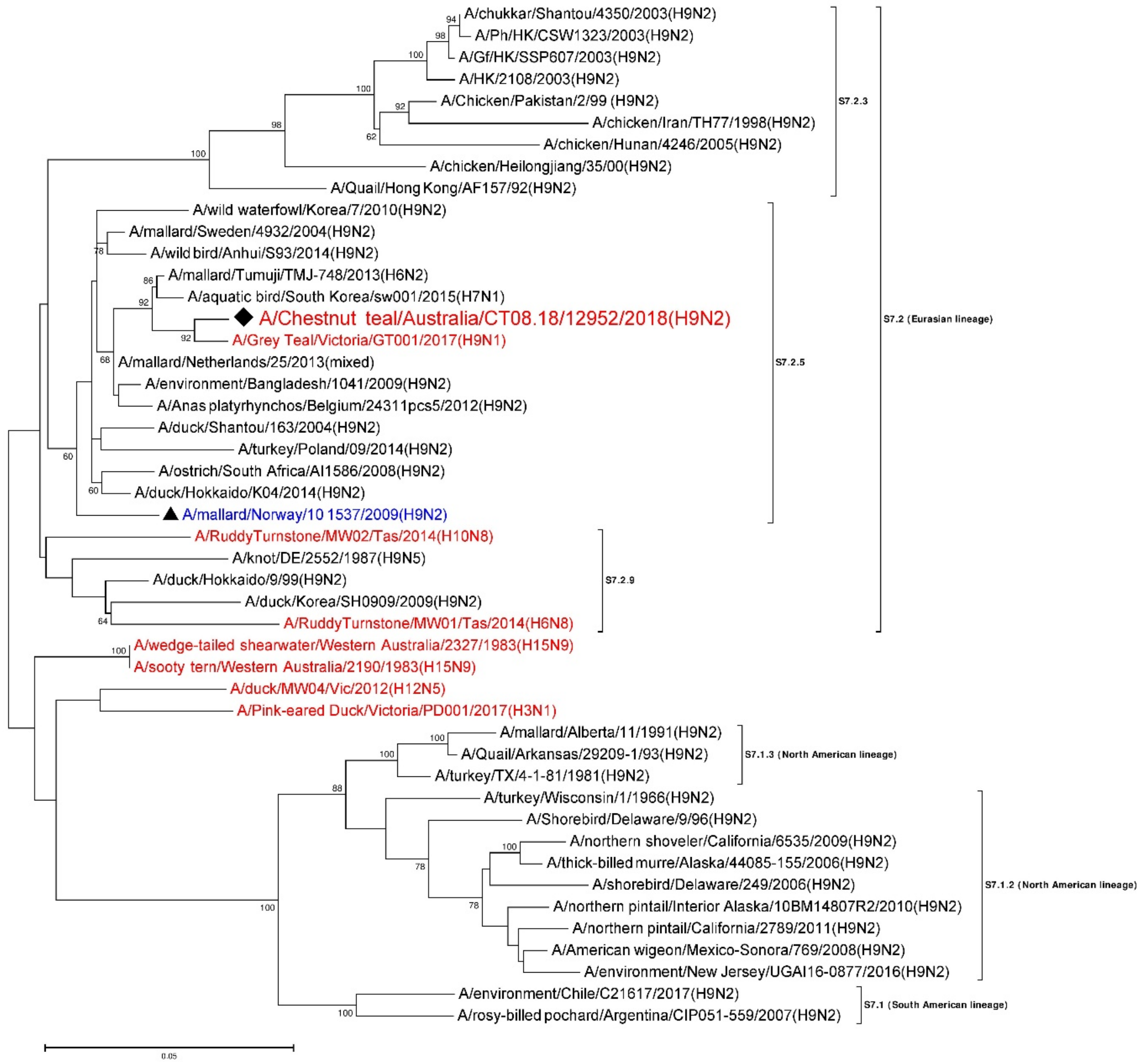
A/chukkar/Shantou/4350/2003(H9N2)
A/Ph/HK/CSW1323/2003(H9N2)
A/Gf/HK/SSP607/2003(H9N2)
A/HK/2108/2003(H9N2)
A/Chicken/Pakistan/2/99 (H9N2)
A/chicken/Iran/TH77/1998(H9N2)
A/chicken/Hunan/4246/2005(H9N2)
A/chicken/Heilongjiang/35/00(H9N2)
A/Quail/Hong Kong/AF157/92(H9N2)
S7.2.3
A/wild waterfowl/Korea/7/2010(H9N2)
A/mallard/Sweden/4932/2004(H9N2)
A/wild bird/Anhui/S93/2014(H9N2)
A/mallard/Tumuji/TMJ-748/2013(H6N2)
A/aquatic bird/South Korea/sw001/2015(H7N1)
A/Chestnut teal/Australia/CT08.18/12952/2018(H9N2)
A/Grey Teal/Victoria/GT001/2017(H9N1)
A/mallard/Netherlands/25/2013(mixed)
A/environment/Bangladesh/1041/2009(H9N2)
A/Anas platyrhynchos/Belgium/24311pcs5/2012(H9N2)
A/duck/Shantou/163/2004(H9N2)
A/turkey/Poland/09/2014(H9N2)
A/ostrich/South Africa/AI1586/2008(H9N2)
A/duck/Hokkaido/K04/2014(H9N2)
A/mallard/Norway/10 1537/2009(H9N2)
S7.2.5
S7.2 (Eurasian lineage)
A/RuddyTurnstone/MW02/Tas/2014(H10N8)
A/knot/DE/2552/1987(H9N5)
A/duck/Hokkaido/9/99(H9N2)
A/duck/Korea/SH0909/2009(H9N2)
A/RuddyTurnstone/MW01/Tas/2014(H6N8)
S7.2.9
A/wedge-tailed shearwater/Western Australia/2327/1983(H15N9)
A/sooty tern/Western Australia/2190/1983(H15N9)
A/duck/MW04/Vic/2012(H12N5)
A/Pink-eared Duck/Victoria/PD001/2017(H3N1)
A/mallard/Alberta/11/1991(H9N2)
A/Quail/Arkansas/29209-1/93(H9N2)
A/turkey/TX/4-1-81/1981(H9N2)
S7.1.3 (North American lineage)
A/turkey/Wisconsin/1/1966(H9N2)
A/Shorebird/Delaware/9/96(H9N2)
A/northern shoveler/California/6535/2009(H9N2)
A/thick-billed murre/Alaska/44085-155/2006(H9N2)
A/shorebird/Delaware/249/2006(H9N2)
A/northern pintail/Interior Alaska/10BM14807R2/2010(H9N2)
A/northern pintail/California/2789/2011(H9N2)
A/American wigeon/Mexico-Sonora/769/2008(H9N2)
A/environment/New Jersey/UGAI16-0877/2016(H9N2)
S7.1.2 (North American lineage)
A/environment/Chile/C21617/2017(H9N2)
A/rosy-billed pochard/Argentina/CIP051-559/2007(H9N2)
S7.1 (South American lineage)

Figure S5

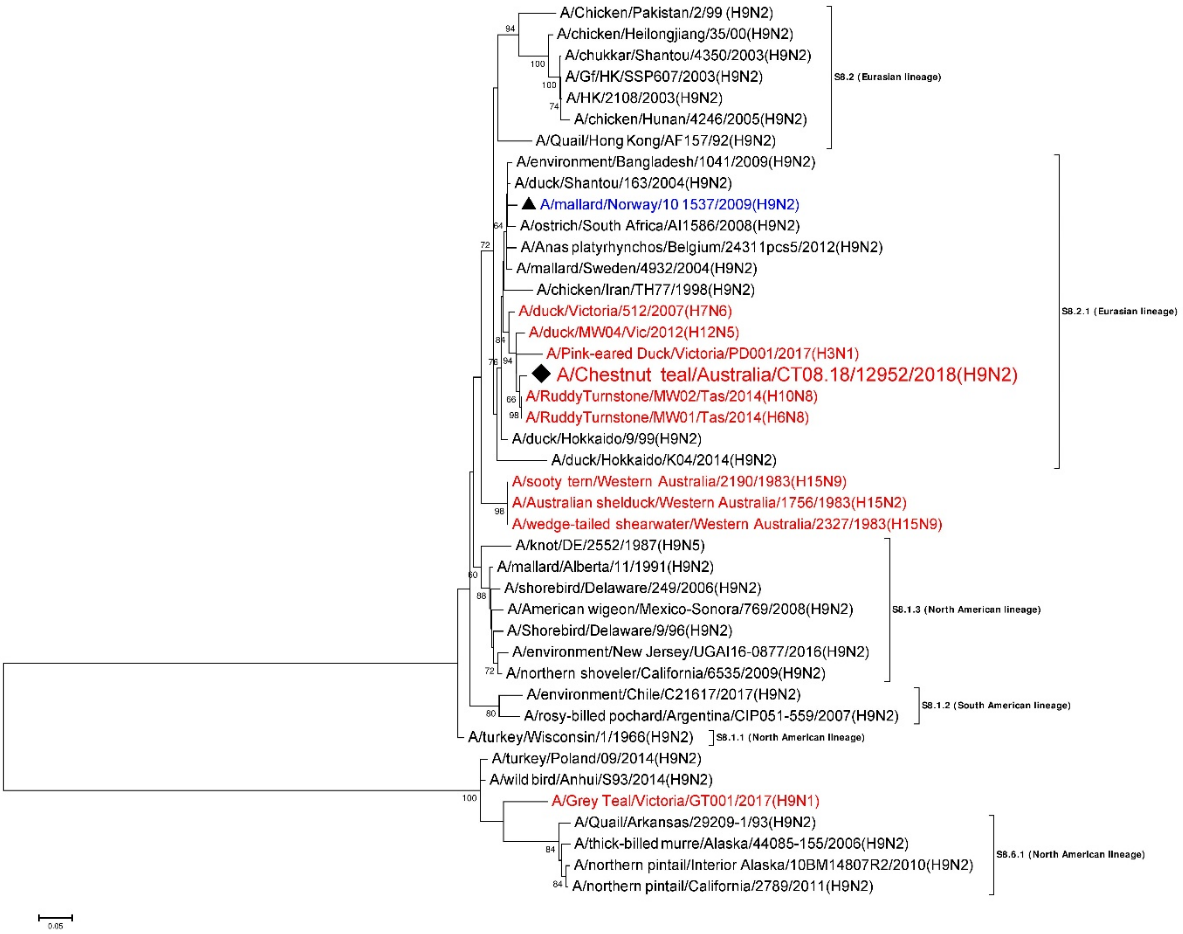

A/Chicken/Pakistan/2/99 (H9N2)
A/chicken/Heilongjiang/35/00(H9N2)
A/chukkar/Shantou/4350/2003(H9N2)
A/Gf/HK/SSP607/2003(H9N2)
A/HK/2108/2003(H9N2)
A/chicken/Hunan/4246/2005(H9N2)
A/Quail/Hong Kong/AF157/92(H9N2)
S8.2 (Eurasian lineage)
A/environment/Bangladesh/1041/2009(H9N2)
A/duck/Shantou/163/2004(H9N2)
A/mallard/Norway/10 1537/2009(H9N2)
A/ostrich/South Africa/AI1586/2008(H9N2)
A/Anas platyrhynchos/Belgium/24311pcs5/2012(H9N2)
A/mallard/Sweden/4932/2004(H9N2)
A/chicken/Iran/TH77/1998(H9N2)
A/duck/Victoria/512/2007(H7N6)
A/duck/MW04/Vic/2012(H12N5)
A/Pink-eared Duck/Victoria/PD001/2017(H3N1)
A/Chestnut teal/Australia/CT08.18/12952/2018(H9N2)
A/RuddyTurnstone/MW02/Tas/2014(H10N8)
A/RuddyTurnstone/MW01/Tas/2014(H6N8)
A/duck/Hokkaido/9/99(H9N2)
A/duck/Hokkaido/K04/2014(H9N2)
S8.2.1 (Eurasian lineage)
A/sooty tern/Western Australia/2190/1983(H15N9)
A/Australian shelduck/Western Australia/1756/1983(H15N2)
A/wedge-tailed shearwater/Western Australia/2327/1983(H15N9)
A/knot/DE/2552/1987(H9N5)
A/mallard/Alberta/11/1991(H9N2)
A/shorebird/Delaware/249/2006(H9N2)
A/American wigeon/Mexico-Sonora/769/2008(H9N2)
A/Shorebird/Delaware/9/96(H9N2)
A/environment/New Jersey/UGAI16-0877/2016(H9N2)
A/northern shoveler/California/6535/2009(H9N2)
S8.1.3 (North American lineage)
A/environment/Chile/C21617/2017(H9N2)
A/rosy-billed pochard/Argentina/CIP051-559/2007(H9N2)
S8.1.2 (South American lineage)
A/turkey/Wisconsin/1/1966(H9N2)
S8.1.1 (North American lineage)
A/turkey/Poland/09/2014(H9N2)
A/wild bird/Anhui/S93/2014(H9N2)
A/Grey Teal/Victoria/GT001/2017(H9N1)
A/Quail/Arkansas/29209-1/93(H9N2)
A/thick-billed murre/Alaska/44085-155/2006(H9N2)
A/northern pintail/Interior Alaska/10BM14807R2/2010(H9N2)
A/northern pintail/California/2789/2011(H9N2)
S8.6.1 (North American lineage)
0.05
